# Exploring nurses’ experiences of psychological distress during care of patients with COVID-19: a qualitative study

**DOI:** 10.1186/s12888-020-02898-1

**Published:** 2020-10-06

**Authors:** Nasrin Galehdar, Aziz Kamran, Tahereh Toulabi, Heshmatolah Heydari

**Affiliations:** 1 Social Determinants of Health Research Center, Lorestan University of Medical Sciences, Khorramabad, Iran; 2 School of Medicine and Allied Medical Sciences, Ardabil University of Medical Sciences, Ardabil, Iran; 3 School of Nursing and Midwifery, Lorestan University of Medical Sciences, Khorramabad, Iran; 4 Social Determinates of health center, Lorestan University of Medical Sciences, Kilomer 4 Khorramabad-Broujerd road Kamalvand, Khorramabad, 6813856967 Iran; 5 Department of Home-based palliative care, ALA Cancer Prevention and Control Center (MACSA), Tehran, Iran

**Keywords:** COVID-19, Nurses, Mental distresses, Qualitative study

## Abstract

**Background:**

COVID-19 infection is a new disease that infects a large number of people, killing a ratio of whom every day in the world. Healthcare staff, especially nurses, experience a great deal of psychological distress during care of COVID-19 patients. Detecting factors that disturb nurses’ mental health during care of these patients can help to reduce their psychological distress. Therefore, this study aimed to explore nurses’ experiences of psychological distress during care of patients with COVID-19.

**Methods:**

The present qualitative research was performed using the conventional content analysis method in Iran from March to May 2020. Participants in this study included the nurses caring for patients with COVID-19, and they were selected based on the purposeful sampling method. The data was collected through 20 phone call interviews and analyzed based on the method proposed by Lundman and Graneheim.

**Results:**

Qualitative data analysis revealed 11 categories including death anxiety, anxiety due to the nature of the disease, anxiety caused by corpse burial, fear of infecting the family, distress about time wasting, emotional distress of delivering bad news, fear of being contaminated, the emergence of obsessive thoughts, the bad feeling of wearing personal protective equipment, conflict between fear and conscience, and the public ignorance of preventive measures.

**Conclusion:**

The data showed that the nurses experienced a variety of psychological distress during care of patients with COVID-19. Through proper planning by authorities, it is possible to manage the risk factors of mental health distress in nurses and improve their mental health status.

## Background

COVID-19 is a newly emerged infectious disease which was first identified in Wuhan, China on December 31, 2019 [1]. The World Health Organization (WHO) declared the disease as a pandemic on March 11, 2020 due to its rapid spread throughout most countries across the globe [2]. Until May 31, 2020, the worldwide numbers of affected and deceased patients with COVID-19 were 6,162,399 and 371,035, respectively. In Iran, until May 30, 2020, the number of people contracting the virus was 148,950 with the death toll standing on 7734 [3]. Around 20% of COVID-19 patients may experience severe symptoms necessitating oxygen therapy or other inpatient interventions, and only 5% of them require admission to the intensive care unit (ICU) [4].

COVID-19, as an emerging disease, is related to the SARS-COV [5] and has many unknown clinical dimensions. Health care workers can play an important role in supporting and helping the patients to rehabilitate [6]. In particular, COVID-19 disease has great effects on healthcare workers and leads to some challenges for this vital part of the society. These problems include an increasing need for medical staff; increasing costs for personal protective equipment (PPE), diagnostic tests, beds and ventilators, as well as rising mortality [7, 8]. Nevertheless, many health care workers are at the risk of contracting the virus and even death, and it is largely impossible to reduce this threat to zero [9]. Due to its unique properties such as high spreading rate, being unknown, and jeopardizing the lives of health care staff, COVID-19 has caused so much confusion and many challenges among health care workers.

Psychological distress including depression and anxiety has been reported in the health staff working at the frontline of fighting COVID-19 amid the outbreak [10–13]. Among health staff, nurses are among the most involved in fighting against the COVID-19. Actually, they regularly are in direct contact with patients from the time of admission to the discharge. Therefore, nurses are highly exposed to psychological distress compared to other health workers during the pandemic [14]. The nurses providing intensive and ongoing health services during previous epidemics had been reported to experience the highest level of job stress and mental distress compared to other treatment staff [15]. Lorestan, as one of the overcrowded provinces of Iran, is located in the western region of the country. Like other parts of the world, the prevalence of the COVID-19 in this province has been increasing since its emergence. Lorestan province has 11 cities with a population of 1,760,000, and its capital is Khorramabad. Eighteen hospitals affiliated to Lorestan University of Medical Sciences are responsible for delivering healthcare to the patients across the province. During the COVID-19 outbreak, Shohaday-e-Ashayer Hospital, as one of the most important hospitals of the province with 350 active beds, was considered as the central site for managing COVID-19 patients. Other counties also allocated parts of their capacities for taking care of patients with COVID-19. According to formal statistics, 1981 nurses are at the frontline of providing care to COVID-19 patients in this province [16]. Therefore, based on the nature of qualitative research which aims to find root problems, and the fact that the researchers are experts on qualitative research methodology, we decided to conduct a qualitative study to explore nurses’ experiences of psychological distress during care of patients with COVID-19. Recognizing the factors affecting nurses’ mental health can help to create a safer workplace for them.

## Methods

This qualitative study was carried out with a conventional content analysis approach.

### Participants

The nurses working in the public hospitals affiliated with Lorestan University of Medical Sciences comprised the study population. The participants were selected by purposeful sampling. Inclusion criteria were at least 2 weeks working experience in taking care of patients with COVID-19 and willingness to participate in the study.

### Collecting data

As there was an immediate need to improve the care for COVID-19 patients, and the fact that face-to-face interviews were risky for both the researchers and nurses, we managed to gather the data through semi-structured in-depth telephone interviews. The calls were made in March and April, 2020. Initially, the characteristics of the nurses working in COVID-19 wards were gathered by looking at their profiles available at the hospitals’ nursing offices. Afterwards, we contacted the participants during their off-times. After explaining the study objectives and acquiring verbal consent from the selected nurses by the first author, a proper time to carry out the interview was agreed with the participant. The duration of the interview was decided based on the nurses’ convenience, tolerance, and working experience. An electronic device was used to record all the interviews. The participants were asked some key questions as “Please describe a day of taking care of hospitalized patients with COVID-19”, and “Please talk about your unpleasant experiences”. The interview continued according to the answers provided by the participant, and based on these answers, more in-depth questions such as “what do you mean?”, and “please explain more about this” were asked.

By choosing probing questions, the researcher directed the interview to achieve the goals of the study. The interviews lasted for 25 to 40 min. Sampling and data collection continued until reaching data saturation and obtaining no new data from the interviews [17].

### Data analysis

Data collection and data analysis were conducted simultaneously. The data was analyzed using Graneheim and Lundman’s content analysis approach [18]. Initially, the interviews were transcribed verbatim. We listened to the interviews and read their transcripts for multiple times to arrive at a general perception of their content. A unit of analysis consisted of a transcript interview whose semantic units included words, sentences, and paragraphs. Semantic units were groups of words or sentences which gave an identical meaning or were relevant to the same concept in some ways. Accordingly, semantic units were condensed and coded. The codes were compared with each other and classified into more abstract categories according to their resemblances. Finally, the categories were compared with each other and sorted into higher-level main categories [18].

### Trustworthiness

In order to determine the accuracy and reliability of the data, the criteria of credibility, confirmability, and transferability were used according to Lincoln and Goba [19]. Member check was used after the formation of primary codes. The authors emailed the text interviews to the participants and asked them to determine whether or not the extracted codes were consistent with their viewpoints and experiences. Finally, and when necessary, data interpretation was corrected based on the participants’ comments. Moreover, we tried to engage a sample with maximum variations in work experience and position, as well as gender and age. We also tried to maintain reflexivity and avoid our own opinion from affecting the study data by precisely reviewing interview transcripts, comparing codes with the raw data, and checking the findings with the participants’ views several times. Peer check was used for conformability, and for this process, code categories were given to three faculty members who were also competent in qualitative research for the external validation of the selection and classification of codes. In term of transferability, the researcher tried for the present of the study context and the participants’ viewpoints with comprehensive detail.

### Ethical considerations

The anonymity and confidentiality of the information and audio files were totally observed. An informed (after explaining the goals and methods) consent was obtained from the participants verbally to prevent the transmission of the disease. The participants were free to withdraw at any moment during the study. The Ethics Committee of Lorestan University of Medical Sciences approved the study protocol (Code: IR.LUMS.REC.1399.006).

## Results

A total of 20 nurses including 15 (75%) women and 5 (25%) men with the mean work experience of 7.25 ± 5.9 years and the average age of 31.95 ± 6.64 years participated in the study (Table 1). Qualitative data analysis revealed 11 categories and 5 sub-categories (Table 2).

**Table 1:** The characteristics of the participants

Participant Number	Gender	Level of Education	Work Experience	Ward	Marital Status	Position
1	Female	Post graduate	4	Emergency	Single	Nurse
2	Female	Bachelor	22	CCU	Married	Head Nurse
3	Female	Student of Post graduate	10	CCU	Married	Nurse
4	Male	Student of Post-graduate	7	Emergency	Married	Nurse
5	Female	Bachelor	20	ICU	Married	Nurse
6	Male	Student of Post graduate	3	Emergency	single	Nurse
7	Female	Student of Post graduate	4	Infectious	Single	Nurse
8	Female	Post graduate	4	Emergency	Married	Nurse
9	Female	Bachelor	1	Emergency	Single	Nurse
10	Female	Bachelor	2	Emergency	Single	Nurse
11	Female	Bachelor	10	ICU	Married	Nurse
12	Female	Bachelor	8	ICU	Married	Nurse
13	Female	Bachelor	14	ICU	Married	Head Nurse
14	Female	Bachelor	4	General	Married	Nurse
15	Female	Student of Post graduate	6	General	Married	Nurse
16	Male	Bachelor	4	ICU	Married	Nurse
17	Female	Bachelor	3	ICU	Single	Nurse
18	Female	Bachelor	12	ICU	Single	Nurse
19	Male	Bachelor	5	ICU	Single	Nurse
20	Male	Bachelor	2	ICU	Single	Nurse

**Table 2:** The categories and sub-categories extracted from the data

Categories	Sub-categories
Death anxiety	Inductive death
	Mortality rate
	Nurses’ inability to help patients
Anxiety due to the nature of the disease	Disease severity
	Disease’s unknown dimensions
Anxiety caused by corpse burial	NA
Fear of infecting the family	NA
Distress about time wasting	NA
Emotional distress of delivering bad news	NA
Fear of being contaminated	NA
The emergence of obsessive thoughts	NA
The bad feeling of wearing PPE^a^	NA
Conflict between fear and conscience	NA
Public ignorance of preventive measures	NA

^a^ Personal protective equipment, *NA* not applicable

### Death anxiety

Data analysis showed that the nurses were subjected to psychological distress witnessing COVID-19 patients’ death. The participants mentioned that the death of patients, especially young ones, was agonizing for them. When patients were suffering from respiratory distress, and the nurses were unable to do anything, this had a huge negative impact on the nurses’ spirits. In this category, there were 3 subcategories including inductive death, mortality rate, and nurses’ inability to help patients.

#### Inductive death

Data analysis showed that many patients had contracted the virus despite strict adherence to preventive precautions. The participants’ experiences showed that the patients hospitalized due to COVID-19 may actually be the victims of others’ carelessness and authorities’ poor decisions. Because of this, the death of COVID-19 patients is much more painful than other patients’ death. In this regard, one of the participants said: “... It is sad that these patients may have been the victims of others’ carelessness ...” [9]. Another participant mentioned“... You feel that patients with COVID-19 may have been infected inadvertently...” [5].

#### Mortality rate

Data analysis showed that one of the nurses’ concerns was the relatively high mortality rate in patients with COVID-19. One participant mentioned: “.... The death of a COVID-19 patient was very stressful for nurses …. especially, the death toll was high at the beginning …. for example, if we had ten patients, we would have expected 3 death and therefore at least 3 announcement of emergency code in that shift... “ [7].

#### Nurses’ inability to help patients

Data analysis showed that the participants regretted that they could do nothing at the time of the patients’ death. In this regard, one of the participants said: “.... It is agonizing to see a person deprived of breath, his heart failing, and you can’t do anything about his suffering .... it sometimes causes me to feel agitated and distressed and becoming really sad and confused about what I’m going to do?” [10]. Another participant says: “... the fact that you can’t do anything in those last moments bothers you a lot, the scenes that I may not forget for the rest of my life ...” [8].

### Anxiety due to the nature of the disease

COVID-19 is a new emerging disease that has a high mortality rate and yet no vaccine or treatment. Under this category, there were two sub-categories of “the disease severity”, and “the disease’s unknown dimensions”.

#### The disease severity

Data analysis showed that COVID-19 is a severe and unpredictable disease. The participants’ experience revealed that at the time of death, the patients had generally acute condition and suffered from severe respiratory distress, rendering a very hard demise. One of the participants said: “ … I myself was caring for a patient with COVID-19, it was really painful to see a person striving to breathe to save himself ... “ [8], another says: “... Dying while feeling suffocated is hard and tormenting ...” [10], and another: “ …. In my opinion, the nature of the disease is beyond what we are teaching and learning now …” [15]. Moreover, data analysis showed that with a high spreading rate and yet no specific treatment, the disease affects many people from all social and age groups. Individuals who have underlying diseases suffer from a more progressive disease, and actually many of them may die. One of the participants noted: “ … we couldn’t even perform intubation as it might cause the disease to progress quickly and the patient to develop lung fibrosis …. we were seeing them dying in front of our eyes, if I didn’t have to, I really wouldn’t go to work ... “ [10]. Addressing the unpredictability of the disease, another participant mentioned: “ .... Sometimes, a patient with a brief lung involvement, and who we didn’t expect at all ..., would die after a while, this was something that really worried us … we were all scared … “ [10]. The same participant continues: “... At first, we thought that, according to reports, only the elderly and people with underlying diseases are affected and may die of the disease, but what we see now is very different, people from all age groups are affected and even die ...” [10]. Another participant mentioned: “.... The nature of the disease was worse than what we imagined ....” [15].

#### The disease’s unknown dimensions

Data analysis showed that many of the disease dimensions were unknown. The disease has currently no vaccine or treatment, and it has a different nature compared with other similar infectious diseases. Furthermore, protocols and guidelines for its management are incomplete. The participants’ experiences revealed that during early days, they were unprepared to protect themselves and provide care services to the patients, which caused them confusion and mental disturbance. One of the participants in this regard said: “…. Not all COVID-19 patients have severe clinical symptoms … there is no correlation between a patient’s death and clinical symptoms … a patient with mild symptoms may die whereas another with severe symptoms recover … the unknown dimensions of the disease are numerous ....” [15]. Another participant stated “.... the disease has no treatment or vaccine.... “ [1].

### Anxiety related to corpse burial

Data analysis also showed that COVID-19 patients’ relatives could not hold funeral and burial ceremonies based on their culture and beliefs for their lost loved ones because the burials of deceased patients should have been done according to health instructions. This itself can negatively affect nurses’ spirit. One participant addressing this issue mentioned that: “.... It is sad that given the type of their death, families cannot manage to choose the type and place of burial and even cannot attend the event...” [9]. Another participant mentioned “.... The condition is not good because they do not have a funeral … and they are not buried in a good circumstance...” [14].

### Fear of infecting the family

Data analysis demonstrated that one of the reasons of the nurses’ mental health problems was fear of infecting their families. The participants’ experience showed that they could not have close contact with their family members because of the risk of being a potential carrier. On their way to return home, the nurses have always had fear and anxiety about being a carrier. Because of this, they may not be able to see their family members for several days.

Data analysis also revealed that nurses who had young children suffered from anxiety and stress because of falling apart from their children. Actually, some of the participants had young children and were compelled to keep a distance from them. One of the participants with maternal concerns said: “I am more worried about my daughter. She is four years old, … and these children have no understanding of the disease, and you can’t say much to them as it may further cause them a lot of distress, … she is always stuck with me, and I’m so stressed … God forbid … generally you have a feeling of guilt that you may infect someone, and that is hard” [11]. Another participant about being separated from her child to prevent disease transmission says: “... I have a little child. It’s been a while since I’ve had a wish to kiss her, this is really hard, when I arrive home, I just have to put on a mask and look at my daughter from a distance, and this is a very difficult situation for us ... “ [4]. Moreover, data analysis showed that the nurses felt responsible for their parents and were concerned about them contracting the virus. The participants’ experiences indicated that some of them had family members with underlying diseases, making them more vulnerable than the general population to COVID-19. This caused the nurses even more distress. One of the participants says: “ … going back to a house where we are not quarantined causes stress about the family, father, and mother because of fear of being a potential carrier … “ [7]. Another participant about the problems of not seeing parents expressed: “... I haven’t seen my mother in two months, she has diabetes and calls me every day and says “come and see me“, but I’m not going to …. “ [10], and another stated: “ …. I see my colleagues suffering from contamination anxiety … …. “ [15].

### Distress about time wasting

Data analysis indicated that nurses spent a lot of time on observing preventive health measures, and this caused nurses to feel tired, distressed, and hesitant to do things on time. The participants’ experience showed that the nurses had to wear protective equipment when they entered the ward, and they also had to disinfect themselves at exit for a while. In addition, they mentioned that the public transportation would refuse to take hospital staff. At home, they also had to spend time washing and disinfecting their bodies. These events generally take a lot of their time and energy. One of the participants says: “... We even enter the changing room one by one, and that means an hour to just change our clothes at the end of our shifts... there is a horror … so we couldn’t get to our work on time. For example, while I finished my shift at 1:30 p.m., I would have arrived at home at 2:30 p.m., and it took me until 3 and 4 p.m. to do my work, and I hadn’t time for the family “ [16].

### Emotional distress for delivering bad news

According to the data analysis, one of the sources of nurses’ distress was the announcement of patients’ death to their families. The experience of the participants showed that informing the families about the death of their loved ones can cause stress in nurses. Families are worried about their loved ones, on the other hand, the prognosis of patients with COVID-19 is not certain. Sometimes, nurses have to explain a patient’s condition to the family, and living with this condition can be stressful for them. One of the participants said: “... We have had mortalities during this period. It was very difficult, especially to inform families ... telling a mother that the condition of her sibling is not good, he is going to die or has died … It’s really very difficult ... “ [18].

### Fear of being contaminated

Data analysis showed that one of the causes of stress in nurses was the fear of being infected. The participants reported that because of the nature of their work, they should keep a close contact with patients, and this can result in mental distress due to the risk of being infected themselves. They also stated that this close relationship and the contaminated environment deterred them from getting adequate sleep and rest, further exaggerating the stress and anxiety. One participant mentioned: “ … This is a situation that I have to take care of people who may infect me at any moment, and then I may transmit the infection to my family, or the fact that I can even die ... these thoughts are coming to me … what will happen to my child, to my life ... “ [10], and another participant mentioned “ …. The virus spreads everywhere, and we may get infected … “ [12].

### The emergence of obsessive thoughts

Data analysis indicated that nurses may develop obsessive thinking during the care of coronavirus patients, so that they see everything and everywhere as contaminated. The participants’ experiences revealed that the nurses refused to eat and drink at work because of obsessive thoughts. One of the nurses noted: “ … There has been an obsession with ourselves and our families … there is the same situation at work too, we used to drink tea or water if we had time, but now we can’t … “ [16]. Another participant reiterated: “.... I mean, I should wash my hands with every garment, that is, I have to do this constantly for every garment ...” [15]. Another participant said: “... I have been obsessed ....” [10].

### The bad feeling of wearing personal protective equipment

Data analysis showed that wearing protective clothes is an unpleasant feeling which has to be experienced by the nurses during each shift. The participants’ experience showed that wearing protective clothes, restrictions in mobility, eating, and drinking, as well as being unknown to others can affect nurses’ mood and lead to extreme fatigue. They noted that nurses’ identity is concealed by wearing protective equipment, which impairs the understanding of the body image and self-esteem. The participants also noted that wearing protective equipment generated a great deal of heat which was hard to bear. One of the participants stated “.... These clothes, these excessive precautions that we have to take because of our job, and the nature of the disease .... we can’t take off or move our masks, glasses, or shields. It puts pressure on us … when your face itches, and you can’t touch it, this is very annoying .... these greatly affect our spirits … “ [16]. Another participant mentioned: “...The fact that they don’t know how I look is a very bad feeling, many of the patients don’t even know if I’m a woman or a man … well, that is a very bad feeling... “ [15].

### Conflict between fear and conscience

The analysis of the data suggested that the nurses had to choose between caring for either themselves or patients, and that created a duality between fear and conscience during patient care. According to the participants’ experience, nurses are the first individuals to contact these patients, and despite being scared and stressed, they take care of these patients because of their conscience. One of the participants mentioned: “.... Along with my fear, I feel pity about why I’m scared and can’t communicate well with patients, it really deters me from communicating with them .... generally, it’s not a good feeling at all, I feel sad for myself that I have to take care of people who may infect me at any moment, and I may infect my family, or I may even die ...” [10]. Another participant noted: “.... This is something between fear and conscience ...” [1].

### Public ignorance of preventive measures

Data analysis showed that one of the causes leading to anxiety in nurses was the public disregarding of preventive precautions. The participants’ experience revealed that despite being aware of the nature of the disease, people continue to ignore health warnings and are actively present in public. Actually, the participants perceived this attitude as an insult to medical staff. Referring to the necessity of a harsher warning to people, one of the participants continued: “ … Why doesn’t the TV show the burial of these patients and the depth of the grave they dig for them, the fact that your family can’t be present at the ceremony, the media should show these to scare people, despite all these recommendations, why don’t people follow and are indifferent to the guidelines.... “ [10]. Also, the data analysis showed that some patients still do not believe that the disease is dangerous, they do not observe health warnings and guidelines when they are dealing with nurses. This augments the fear, anxiety, and the risk of nurses becoming infected. One of the participants mentioned: “ … All the times there are coughs and sneezes, for example, you want to puncture a vein, they do not observe and cough and sneeze over our face .... “ [2].

## Discussion

In this study, it was shown that the nurses experienced a great deal of psychological problems while providing care services for patients with COVID-19. In line with the findings of this study, many other studies have reported high levels of psychological distress among nurses during outbreaks [10–12]. Also, a systematic review and meta-analysis has shown a high prevalence of mental disturbances such as anxiety and depression among healthcare workers during the COVID-19 pandemic [13]. Our findings were in line with nurses’ experiences in previous epidemics of infectious diseases such as SARS, MERS-Cov, Ebola, and H1N1 influenza during which nurses developed mental distresses such as loneliness, anxiety, fear, fatigue, sleep disorders, and other physical health problems [20–22].

In this study, nurses experienced anxiety and distress due to COVID-19 patients’ death. Death due to the COVID-19 disease may be unsettling as in many cases, the patient plays a non-determinant role in contracting the infection. In some cases, patients might even have good adherence to health behaviors, yet they get infected because of others’ ignorance about health recommendations. The death of these patients can negatively affect the mental health of nurses. Death anxiety is a multidimensional construct involving cognitive, emotional, and experiential aspects [23]. In line with the findings of the current study, other studies have shown that nurses may not be prepared to communicate with dying patients and their families [24, 25], and they may become anxious observing dying patients [26].

Furthermore, nurses’ attitudes toward death may affect their empathy for patients [27], the quality of care [28], and the ways exploited to cope with the stress of patients’ death [29]. In this regard, a study showed that nurses did not receive effective training to provide care for dying patients [25].

The results of studies have shown that holding educational programs about death can reduce death anxiety in nurses and improve the quality of nursing care in critical situations [28]. Training programs for the management of death anxiety should include psychology of bereavement, societal viewpoints on death, symptom management, communication skills, as well as supportive interventions [28]. It seems that in the time of COVID-19 outbreak, holding virtual workshops to reduce death anxiety in nurses can be effective.

Considering the contagious nature of the disease, nurses are forced to live away from their families while they care of COVID-19 patients. In the present study, the nurses reported to have the anxiety of being separated from their children and parents and were worried about the possibility of transmitting the disease to their family members. This finding was consistent with the results of previous similar studies [30, 31]. It seems that providing the possibility of visual communication with family members through social media networks, as well as face-to-face meeting while watching necessary health protocols can minimize the emotional burden of this condition in nurses.

In this study, the nurses also reported a fear of being infected with the virus. This was consistent with similar previous studies [31, 32]. This fear increases anxiety and stress among healthy people and exacerbates the symptoms of mental distress during epidemics [33]. From the sources contributing to this fear are protective equipment shortage and the lack of information and skills required to protect oneself. Providing enough personal protective equipment, holding training courses, and providing psychological support for the nurses taking care of COVID-19 patients can help to reduce the anxiety related to such fears.

Wearing PPE was reported to be unpleasant, hard, and tedious for the nurses. As we have seen the evidence in social media, due to prolonged usage of personal protective equipment, traces of scars have been seen on nurses’ faces. Also, using these covers prevents nurses from face to face and eye contact with colleagues as well as patients [34]. This can even lead to more exhaustion of nurses [35], as well as their hunger and thirst. It seems that these issues can be resolved by considering rest intervals during shifts and shortening working hours, which allow nurses to refresh during rest by drinking, eating, taking a shower, and going to the bathroom.

The emotional distress of delivering bad news was another stressful experience reported by our participants in this study. Telling bad news is one of the difficulties of the medical profession [36], and having skills to impart such news is one of the most essential communication skills required for health staff. Therefore, the principles of effective communication with patients and their families should be carefully considered in times of crises such as the COVID-19 pandemic [37]. Nurses need the abilities to inform bad news and manage tense situations, which cause themselves to experience a lower stress and victims’ families to accept bad news with more tolerance and patience.

A conflict between fear and conscience was another experience reported by the nurses in this study. Despite that nurses see themselves in danger, they try to carry out their duties with good quality. As a professional mission, it is expected from health care providers to deliver health services with maximum honesty and courage. In this regard, feeling safe and receiving support can boost the quality of performing this duty by nurses [37]. During pandemic crises, nurses are exposed to life-threatening risks due to their high work-loads, the lack of resources and protective equipment, a high probability of contracting the disease, and generally unknown nature of the disease [34]. Despite shortages in resources and other mentioned limitations, they cannot refuse to provide services and fulfill their professional mission. Therefore, they experience an internal pressure regarding professional ethics [32, 38].

Ignoring health protocols by patients was another experience of the nurses in this study. Several evidences indicate that people have low adherence to self-quarantine and preventive recommendations [39, 40]. Overlooking these recommendations can increase the risk of propagating the disease throughout the community. In this regard, it is necessary to direct people to accept social responsibilities about the health of other members of the society and present health recommendations to them based on behavior-changing theories. Mass media networks by showing entertaining programs should also encourage people to stay at home. Of course, one of the major concerns of people, that leads them to ignore health instructions, is the economic crisis. Therefore, with financial support of poor people and providing fun programs through mass media, it is possible to encourage people to stay at homes and adherence to health instructions.

About the limitations of this study, it can be said that a qualitative study should be conducted through relatively long face to face interviews in order to obtain a stronger rigor of data, but in this study, the data was collected in a short time. Besides, because of the urgent situation and to reduce the risk of infection for both the interviewee and interviewer, the data was collected via phone interviews. Nevertheless, the validity of the data was ensured using alternative methods.

## Conclusion

The nurses’ experiences in this study showed that they endured a great deal of psychological distress during care of patients with COVID-19. The sources of such distress were related to patients’ death, the disease’s unknown dimensions, the atmosphere of the working environment, professional commitments, and individual characteristics. During outbreaks of diseases and/or natural disasters and in accordance with their professional duties and missions, nurses actively provide health services for people from the beginning of crises. In order to boost nurses’ mental well-being, there is a need for support from decision and policy makers and governments during and after such events. If no support is provided, nurses’ workforce will be degraded, which can deliver the health system vulnerable to the crisis. Paying attention to the experiences of nurses during the coronavirus pandemic is a prerequisite for providing health cares and improving their quality. For maximum-quality health care services during pandemics, governments and health systems should minimize nurses’ mental burden by delivering adequate personal protection equipment, strengthening mutual support among health team members, providing psychological counseling, and delivering timely information and educational supports. Encouraging people to adhere to health instructions and to stay at home can reduce the disease propagation and the workload of nurses and improve their mental health. Holding training workshops on how to cope with the current crisis, encouraging nurses to listen to each other’s concerns, and preventing negative news from being spread among health care workers can also improve nurses’ mental health. It is recommended to conduct more research on how to improve nurses’ mental health during the COVID-19 outbreak.

## Data Availability

The datasets generated during and/or analyzed during the current study are available from the corresponding author on request.

## Abbreviations

COVID-19: Coronavirus disease- 2019
MERS-COV: Middle East Respiratory Syndrome coronavirus
SARS-COV: Severe acute respiratory syndrome coronavirus
WHO: World Health Organization
